# PLGA nanoparticles for the oral delivery of nuciferine: preparation, physicochemical characterization and *in vitro*/*in vivo* studies

**DOI:** 10.1080/10717544.2016.1261381

**Published:** 2017-02-06

**Authors:** Ying Liu, Xin Wu, Yushuai Mi, Bimeng Zhang, Shengying Gu, Gaolin Liu, Xiaoyu Li

**Affiliations:** 1 Department of Clinical Pharmacy,; 2 Department of General Surgery, and; 3 Department of Acupuncture and Moxibustion, Shanghai General Hospital, Shanghai Jiao Tong University School of Medicine, Shanghai, PR China

**Keywords:** Nuciferine, PLGA, nanoparticles, bioavailability, lipid lowering

## Abstract

This article reports a promising approach to enhance the oral delivery of nuciferine (NUC), improve its aqueous solubility and bioavailability, and allow its controlled release as well as inhibiting lipid accumulation. NUC-loaded poly lactic-co-glycolic acid nanoparticles (NUC-PLGA-NPs) were prepared according to a solid/oil/water (s/o/w) emulsion technique due to the water-insolubility of NUC. PLGA exhibited excellent loading capacity for NUC with adjustable dosing ratios. The drug loading and encapsulation efficiency of optimized formulation were 8.89 ± 0.71 and 88.54 ± 7.08%, respectively. NUC-PLGA-NPs exhibited a spherical morphology with average size of 150.83 ± 5.72 nm and negative charge of −22.73 ± 1.63 mV, which are suitable for oral administration. A sustained NUC released from NUC-PLGA-NPs with an initial exponential release owing to the surface associated drug followed by a slower release of NUC, which was entrapped in the core. In addition, ∼77 ± 6.67% was released in simulating intestinal juice, while only about 45.95 ± 5.2% in simulating gastric juice. NUC-PLGA-NPs are more efficient against oleic acid (OA)-induced hepatic steatosis in HepG_2_ cells when compared to naked NUC (n-NUC, *p < 0.05). The oral bioavailability of NUC-PLGA-NPs group was significantly higher (**p < 0.01) and a significantly decreased serum levels of total cholesterol (TC), triglycerides (TG) and low-density lipoprotein cholesterol (LDL-C), as well as a higher concentration of high-density lipoprotein cholesterol (HDL-C) was observed, compared with that of n-NUC treated group. These findings suggest that NUC-PLGA-NPs hold great promise for sustained and controlled drug delivery with improved bioavailability to alleviating lipogenesis.

## Introduction

The excess lipid accumulation within hepatocytes followed by subsequent inflammation was eventually developed into liver damage (Woods et al., 2015). Several alkaloid extractions of lotus leaf, a traditional Chinese medicinal herb, have been shown to possess therapeutic potentials for obesity and atherosclerosis via accelerating lipid metabolism, reducing triglyceride accumulation in adipocytes and inhibiting the absorption of lipids and carbohydrates (Wang et al., 2015). Nuciferine (NUC), an aporphine alkaloid, is thought to be responsible for the active ingredient of *Nelumbo nucifera*, which possessed a broad range of pharmacological activities containing ameliorating hyperlipidemia, lowering cholesterols, dilating vessels, stimulating insulin secretion and improving hepatic lipid metabolism. In our previous study, we successfully demonstrated that NUC owns potential of attenuating or inhibiting lipid accumulation and inflammation and its possible underlying mechanism (Zhang et al., 2015).

The clinical application of NUC has been hampered due to its bioavailability. We have calculated the values of absolute bioavailability according to oral doses of 2.0, 5.0 and 10.0 mg/kg and intravenous (IV) administration of 0.2 mg/kg NUC in rats. The results were (3.8 ± 1.4), (4.2 ± 1.3) and (3.9 ± 1.0)%, respectively (Gu et al., 2014). We concluded the low absolute bioavailability was related to poor absorption, rapid metabolism and rapid systemic elimination.

Oral administration was suggested to own the maximum patient compliance, while the poor bioavailability is a conundrum not yet solved (Pathak & Raghuvanshi, 2015). In order to reach therapeutic level, drugs with poor oral bioavailability are administered at increased dose or designed as a novel delivery system that can exhibit improved pharmacokinetic profiles. Increased dose may induce the poor compliance, wastage of drug, which is not economical bearing, especially the expensive drugs and most importantly the adverse effects. Pharmaceutical manufacturers thus optimize the drug molecule, which has gradually evolved into the progress of micro-sized and nano-sized medication (Cherniakov et al., 2015).

Nanomedicine is rapidly gaining recognition for enhancing the bioavailability of drugs in their adjustable dosage formulations, especially highly hydrophilic drugs (Ozeki & Tagami, 2013). Poly (lactic-co-glycolic acid) (PLGA) is a copolymer of polylactic acid (PLA) and polyglycolic acid (PGA) that has been approved for clinical application by the US Food and Drug Administration (FDA) (Zhao et al., 2016). PLGA has excellent biocompatibility and degradability in physiological environments and the biodegradation products (lactic acid and glycolic acid) are naturally occurring metabolites. By virtue of these advantages, PLGA has been used as an efficient carrier for food and drug delivery. Shi et al. employed mono-PEGylation combined with PLGA delivering Radix Ophiopogonis polysaccharide, a poorly water-soluble macromolecule, to solve the problem of its relatively short half-life *in vivo* (Shi et al., 2014). Chen et al. developed a biodegradable micellar system composed by glycyrrhetinic acid and PLGA to encapsulate Tanshinone IIA targeting hepatocellular carcinoma (Chen et al., 2016).

In this study, we perfected and fine-tuned the bioavailability of drug by loading water insoluble NUC in PLGA based on a solid/oil/water (s/o/w) emulsion technique. The NUC-loaded poly lactic-co-glycolic acid nanoparticles (NUC-PLGA-NPs) were characterized with regard to particle size, zeta potential, morphology and encapsulation efficiency and drug loading as well as the *in vitro* release of NUC. Both the effects of n-NUC and NUC-PLGA-NPs against OA-induced hepatic steatosis in HepG_2_ were studied. NUC’s oral bioavailability and lipid lowering *in vivo* were also evaluated in male Sprague Dawley (SD) rats.

## Materials and methods

### Materials

Poly (D,L-lactic-co-glycolic acid) (PLGA, molar ratio of D,L-lactic to glycolic acid, 50:50, MW = 30 KDa) was purchased from Jinan Daigang Biomaterial Co., Ltd (Shandong, China); bovine serum albumin (BSA) was acquired from Amresco (Solon, OH); NUC, DIL, coumarin-6, Oil-red-O (ORO), oleic acid (OA), and vitamin E were purchased from Sigma-Aldrich (St. Louis, MO). Dulbecco’s Modified Eagle’s Medium (DMEM), fetal bovine serum (FBS) and penicillin–streptomycin solution (5 KU/mL) were purchased from Thermo Fisher Scientific (Waltham, MA). A Cell Counting Kit-8 (CCK8) was obtained from Dojindo Molecular Technologies Inc. (Tokyo, Japan). Annexin V-PE/7AAD Apoptosis Detection Kit was obtained from BD Pharmingen (San Diego, CA). All other chemicals used were analytical grade and organic solvents were high performance liquid chromatography (HPLC) grade.

### Cell culture

Human hepatocellular carcinoma (HCC) cells HepG_2_ was purchased from Cell Culture Center of Shanghai Institutes of the Chinese Academy of Sciences (Shanghai, China). HepG_2_ were cultured in high glucose DMEM containing 10% FBS and 1% penicillin–streptomycin under an incubator with 5% CO_2_ at 37 °C.

### Animals

Adult male SD rats weighing 220–240 g were purchased from SLAC Laboratory Animal Co. Ltd (Shanghai, China). Rats were maintained at stable temperature (23 ± 2 °C) and humidity (45–55%) under a 12-h light/dark cycle. All animal experiments were in accordance with the Animal Care and Use of Laboratory Animals of Shanghai General Hospital and followed the guidelines of the Animal Welfare Act.

### Preparation of NUC-PLGA-NPs

NUC-PLGA-NPs were prepared according to a s/o/w emulsion technique with moderate modification (Perez et al., 2002). Briefly, PLGA was dissolved in dichloromethane and acetone at a volume ratio of 3:2 to acquire a uniform PLGA solution (10 mg/mL). Free NUC was added to the PLGA solution and sonicated at 100 W for 60 s to produce the s/o primary emulsion. The acquired emulsion was then added to 4 mL of BSA solution (1% w/v) and again sonicated at 100 W for 30 s twice time to generate the final s/o/w emulsion. To disperse the final s/o/w emulsion, 30 mL of distilled water was added followed by 4 h of magnetic stirring for the removal of residual organic solvents. The NPs were finally acquired after centrifugation at 14 000 rpm for 30 min and the supernatant was discarded. The obtained NUC-PLGA-NPs were washed with distilled water three times before lyophilization with vacuum freeze dryer (VirTis, Stone Ridge, NY) under −50 °C. The freeze-dried powder was stored at 4 °C for future use.

### Characterization of NUC-PLGA-NPs

#### Encapsulation efficiency and drug loading

To evaluate the encapsulation efficiency ratio (EE%) and drug loading ratio (DL%), NUC-PLGA-NPs with various amounts of NUC were prepared as described in the part of preparation of NUC-PLGA-NPs. NUC levels in the PLGA nanoparticles were assayed by HPLC. The EE% of the NUC-PLGA-NPs was calculated as NUC encapsulated in respect to the feeding NUC (wt%/wt%) to prepare the NUC-PLGA-NPs and the DL% was expressed as the amount of NUC encapsulated in respect to the NUC-PLGA-NPs (wt%/wt%). To disrupt NPs structure, 5 mg lyophilized NUC-PLGA-NPs were dissolved in 1 mL methanol and vortexed with ultrasonic waves for 10 min to ensure encapsulated NUC release. Then, the samples in acetonitrile were centrifuged at 10 000 rpm for 10 min and supernatant (100 μL) gathered was diluted to 1 mL for encapsulation and loading detection as previously mentioned.

#### Fourier transform infrared spectroscopy (FTIR)

FTIR method was used to identify the functional groups related to structure and composition via absorption spectrum. The infrared spectra of the n-NUC, PLGA and NUC-PLGA-NPs, prepared as KBr discs, were measured over the range of 4000–400 cm^−1^.

#### Differential scanning calorimetry (DSC)

Thermograms of n-NUC, PLGA and NUC-PLGA-NPs were obtained with a differential scanning calorimeter (DSC 200 F3 Maia, Germany) and calibrated using a pure indium sample. Different samples (2–3 mg) were placed in aluminum pan heated up at 10 °C/min within 0–550 °C under dry nitrogen, respectively.

#### Size and zeta potential

The particle size and zeta potential of drug-free NPs and NUC-PLGA-NPs were determined by dynamic light scattering (DLS) using a Zetasizer Nano-ZS90 (Malvern Instrument, Worcestershire, UK). All samples were diluted in distilled water and equilibrated for 30 min before measurement. Each sample was analyzed in triplicate.

#### Transmission electron microscopy (TEM)

The morphology and size of the dried NUC-PLGA-NPs was measured using a TEM. A drop of NUC-PLGA-NPs suspension was placed onto a 200 mesh copper grid and dried at room temperature (RT) for view.

### Release kinetics *in vitro*

A fixed weight of NUC-PLGA-NPs (100 mg) was dispersed in simulated gastric fluid (0.2% w/v NaCl in 0.7% v/v HCl at a pH of 1.2) and simulated intestinal fluid (pH 6.8) without enzymes, respectively. The solution was divided into 24 Eppendorf tubes with continuous shaking at 120 rpm at 37 °C. Aliquots (100 μL) were withdrawn and centrifuged at 10 000 rpm for 10 min after predetermined time intervals (1, 2, 4, 8, 12, 24, 48, 72, 96, 120, 144 and 168 h). After removing the supernatant, the pellet was resuspended with 1 mL methanol for HPLC detection.

### Cellular uptake

For confocal laser scanning microscopy (CLSM, Leica Microsystems, Wetzlar, Germany) imaging, coumarin-6 (green fluorescence) was loaded as described in the section of preparation of NUC-PLGA-NPs, apart from the change of NUC to a hydrophobic fluorescent probe coumarin-6. HepG_2_ cells (1 × 10^4^ cells/well) were seeded into confocal dish for 24 h. After incubated with free coumarin-6 or coumarin-6-loaded NPs for 4 h, cells were incubated with DIL (red fluorescence for general cell membrane labeling) for 20 min and then treated with Hoechst for staining of nucleus.

### *In vitro* cytotoxicity assay

Cell proliferation of HepG_2_ treated with OA (0.04 mM as our previously used) and blank PLGA, n-NUC and NUC-PLGA-NPs supplemented with OA were measured using the CCK8 assay kit according to the manufacturer’s instructions. The concentration of NUC used in n-NUC and NUC-PLGA-NPs was 0.05 mM as previously used (Zhang et al., 2015). The untreated cells were selected as control. Briefly, 10  μL CCK8 solution was added to each well (100-μL medium) incubated with different formulations described above and incubated for additional 1.5 h at 37 °C. The absorbance was recorded at 450 nm with a microplate reader (Thermo Scientific, Rockford, IL).

### Cell apoptosis analysis

Cells apoptosis of HepG_2_ treated with OA and PLGA, n-NUC and NUC-PLGA-NPs supplemented with OA were measured using the Annexin V-PE/7AAD Apoptosis Detection Kit following the manufacturer’s instructions. The untreated cells were selected as control. All samples were analyzed by flow cytometry (BD Accuri C6; BD Biosciences, San Jose, CA).

### Oleic acid-induced hepatic steatosis

OA was selected to induce *in vitro* model of hepatic steatosis as previously described with slight modifications. Briefly, HepG_2_ cells were incubated with OA (0.04 mM) for 24 h to induce cellular steatosis. To determine the effect of NUC on OA-induced steatosis, cells were incubated with PLGA, n-NUC, NUC-PLGA-NPs and vitamin E (0.025 mM, selected as positive control) 24 h before treatment with 0.04 mM OA. The concentration of NUC used in n-NUC and NUC-PLGA-NPs was 0.05 mM and the untreated cells were selected as control. Cells were gently washed with phosphate buffered saline (PBS) three times and fixed using 4% paraformaldehyde at RT for 20 min. Subsequently, cells were washed with PBS three times, stained with freshly prepared working solution of ORO (diluted in double-distilled H_2_O at volume ratio of 3:2) for 30 min at RT, and redyed for 30 s  in hematoxylin staining solution. Finally, cells were washed with PBS five times before examined under EVOS microscope (Thermo Fisher Scientific, Waltham, MA). The intracellular lipid accumulation was quantitated by Image-Pro Plus 6.0 (Media cybernetics Inc., Bethesda, MD).

### Pharmacokinetic

For the pharmacokinetic study, 18 male SD rats weighing 200–240 g were randomly assigned to three groups (*n* = 6) for IV administration of 0.2 mg/kg n-NUC, oral administration of n-NUC or NUC-PLGA-NPs at NUC concentration of 5 mg/kg, respectively. Blood samples (∼200 μL) were collected into heparinized tubes at 2 min, 5 min, 15 min, 30 min, 45 min, 1 h, 2 h, 3 h, 4 h, 5 h and 6 h after IV administration and at 5 min, 15 min, 30 min, 45 min, 1 h, 2 h, 3 h, 4 h, 6 h, 9 h and 12 h after oral administration. The blood samples were immediately centrifuged at 8000 rpm for 5 min. The plasma was separated by centrifugation and stored at −80 °C before analysis by HPLC-MS/MS, of which parameters were set as previously used (GU et al., 2014).

The peak concentration (C_max_) and the time of peak concentration (t_max_) were observed from experimental results, and pharmacokinetic parameters such as half-life time (t_1/2_), total area under curve (AUC_t_) were processed by non-compartmental analysis using WinNonlin software (Certara, Princeton, NJ, USA). The absolute bioavailability (F_abs_) was calculated as follows: F_abs_ (%) = (AUC_ig _×Dose_iv_)/(AUC_iv _×Dose_ig_) × 100%, while the relative bioavailability (F_rel_) was calculated by the following formula: F_rel_ = (AUC_A _×Dose_B_)/(AUC_B _×Dose_A_) × 100%.

### The effect of NUC on male Sprague Dawley rats with high fat diet-induced hyperlipidemia

After adaptive feeding for 7 days, rats were randomly distributed into four groups.

Group I: Normal control (NC) group fed a regular laboratory diet.

Group II: Hyperlipidemia control (HC) fed high-fat diet (HFD, consisting of 68.5% standard laboratory chow, 15% carbohydrate, 10% lard, 5% yolk powder, 1% cholesterol, and 0.5% sodium cholate.

Group III: Rats fed HFD supplemented with n-NUC (10 mg/kg/day).

Group IV: Rats fed HFD supplemented with NUC-PLGA-NPs at NUC of 10 mg/kg/day.

After 2, 4, 6 and 8 weeks of treatment, blood was collected by retro-orbital puncture and plasma was separated by centrifuged at 1000 rpm for 15 min. Total cholesterol (TC), triglycerides (TG), high-density lipoprotein cholesterol (HDL-C) and low-density lipoprotein cholesterol (LDL-C) were assayed by using kits purchased from Bio-Swamp (Shanghai, China).

### Statistical analysis

The data were expressed as the mean ± SD. Statistical analysis was performed using Student’s t-test and one-way ANOVA. A value for *p < 0.05 was considered statistically significant.

## Results and discussion

### Preparation and characterization of NPs

Oral administration is widely used due to its low-associated costs and high-patient compliance as compared to other routes like IV, intramuscular and subcutaneous injection. However, oral drugs with poor aqueous solubility and sensitive to degradation are unable to reach the minimum effective concentration exhibiting therapeutic effect. Nanotechnology-based drug delivery system has shown to improve solubility and shield against digestive enzymes and pH changes, among which PLGA is particularly interesting for oral drug delivery due to its biocompatibility, degradability in physiological environments, commercial availability of various grades and the safe biodegradation products. NUC, a raw material in Chinese medicinal herb, which is an aporphine alkaloid extracted from lotus leaves contributing to lipid metabolism. In this study, NUC powder was suspended in the PLGA solvent composited of dichloromethane and acetone at a volume ratio of 3:2. Basing on the emulsion encapsulation techniques, this suspension was emulsified in an aqueous solution containing BSA. Organic solvent was then evaporated, after which nanoscaled products were finally washed and lyophilized.

#### Encapsulation efficiency and drug loading

**Figure 1.**
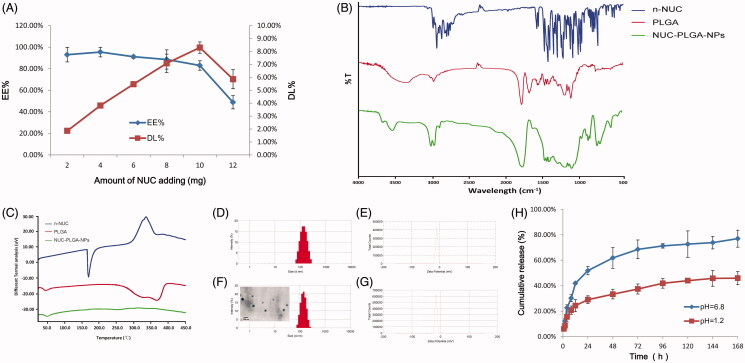
(A) Encapsulation efficiency (EE%) and drug loading (DL%) of nanoparticles based on various amount of NUC adding (mean ± SD, *n* = 3). FTIR spectra (B) and DSC curves (C) of NUC, PLGA and NUC-PLGA-NPs. Size distribution (D) and zeta potential (E) of PLGA. Size distribution and a representative TEM image (F) and zeta potential (G) of NUC-PLGA-NPs. (H) *In vitro* release of NUC from NUC-PLGA-NPs in simulating gastric fluid at pH 1.2 and in simulating intestinal fluid at pH 6.8 over a period of one week (mean ± SD, *n* = 3).

The EE% and DL% of NUC-PLGA-NPs at various NUC feeding were shown in Figure 1(A). With increasing concentrations of NUC, the EE% decreased, while the DL% increased first and then decreased sharply. The decrease of EE% might be attributed to excessive drug, which hampered the overall stability of the formed NUC-PLGA-NPs after PLGA reached the saturation solubility in the polymer matrix (Xin et al., 2010). Fortunately, the DL% could reach 8.89 ± 0.71% along with EE% at 88.54 ± 7.08% when adding 8 mg NUC. Based on these results, we optimized the feeding amount of NUC at 8 mg when 100 mg PLGA was used in the following experiments.

#### FTIR spectroscopy

FTIR spectra of n-NUC, PLGA and NUC-PLGA-NPs were shown in Figure 1(B). The spectrum of PLGA showed a characteristic peak at 1757 cm^−1^ which indicated unconjugated carbonyl (C=O) stretching (Zabelin et al., 2016). The NUC showed peaks in the range of 1450–1600 cm^−1^, which belonged to frame vibration of benzene ring. NUC-loaded NPs showed peak at 1680 cm^−1^ corresponding to PLGA and characteristic peak of benzene ring with moderate peak shifting, which confirmed the encapsulation of NUC in PLGA.

#### DSC

DSC thermograms of n-NUC, PLGA and NUC-PLGA-NPs are shown in Figure 1(C). The drug showed a sharp peak at 165 °C consistent with its melting point of crystalline regions, while PLGA exhibited an endothermic relaxation peak at 50 °C corresponding to its glass transition temperature (Gidwani & Vyas, 2016). Owing to the fact that PLGA is amorphous in nature, there was no distinct melting point. NUC-PLGA-NPs exhibiting the similar relaxation peak as PLGA and the characteristic peak of n-NUC were not viewed in NUC-PLGA-NPs, indicating that drug was encapsulated by PLGA in an amorphous or disordered-crystalline phase of a molecular dispersion or a solid solution state in the polymer matrix.

#### Size and zeta potential

After re-dispersion in deionized water, drug-free NPs and NUC-PLGA-NPs were characterized concerning average size, width of distribution and zeta potential. A typical DLS size and zeta profile on the distribution of NPs and NUC-PLGA-NPs were shown in Figure 1(D, E, F, and G), respectively. The average size of empty NPs was 130.58 ± 5.36 with polydispersity index (PDI) of 0.26 ± 0.028, while an increase size of 150.83 ± 5.72 with PDI of 0.24 ± 0.025 was observed for NUC-PLGA-NPs, probably for the presence of NUC entrapped into the core of PLGA. Nanoparticles of this size not only can be internalized by endocytosis but also are large enough to be maintained in circulation for a long period (Anselmo & Mitragotri, 2016). More negative (−) or positive (+) electricity prevented the particles from aggregation due to the repelling interaction (Honary & Zahir, 2013; Gossmann et al., 2015). Zeta potential values of drug-loaded NPs and unloaded ones were −23.45 ± 1.54 and −22.73 ± 1.63 mV, respectively, indicating the stability of NUC-PLGA-NPs in suspension. NUC-PLGA-NPs exhibit spherical morphology with low tendency of agglomeration. The TEM-observed size was around 100 nm smaller than DLS-calculated size, because the latter size refer to the hydrodynamic diameter of the nanoparticles with a polymer layer, while the former one represented only the core of the nanoparticles (Han et al., 2016).

### Release kinetics *in vitro*

*In vitro* release of NUC from NUC-PLGA-NPs was determined by imitating digestion conditions in gastric and intestinal juice, respectively. As shown in Figure 1(H), NUC release fast within the first 12 h both in simulating gastric fluids and intestinal fluids, may be due to the presence of loosely bound NUC on or near the surface of particles (Sun et al., 2015), followed by a very slow release up to 45.95 ± 5.2% in artificial gastric fluids and sustained and prolonged release up to 77 ± 6.67% in artificial intestinal fluids. Acidic environment is responsible for the protonation of the carboxyl groups of PLGA and the aggregation of the nanoparticles, which shapes into a stable structure to limit NUC release from the nanocomposites. While the increased pH in artificial intestinal juice triggered NUC release from the NUC-PLGA-NPs due to the increased water absorption of GA group, which induced the penetration of the water toward the core of nanocomposites. On the basis of the results above, we concluded that minimal amounts of NUC could be released from the nanocomposites in the stomach when given orally, while the residues could achieve a sustained release and effective treatment.

### Cellular uptake

**Figure 2.**
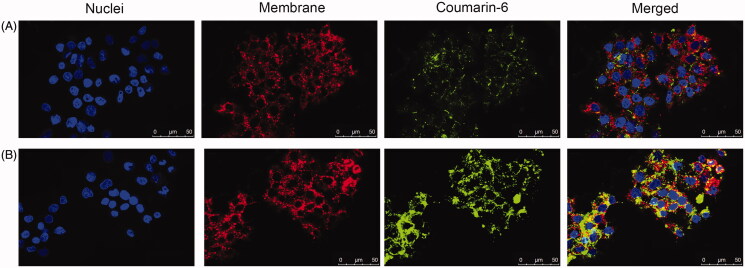
Cellular uptake of free coumarin-6 (A) and coumarin-6-loaded NPs (B) against HepG2. Scale bar: 50 μm.

To illustrate the intracellular delivery of NUC by PLGA, coumarin-6 (green fluorescence) was encapsulated into NPs on account of the hydrophobic property as NUC. In Figure 2, CLSM images show cell membranes stained in red with DIL and general nucleus labeled by Hoechst (blue fluorescence). Free coumarin-6 could hardly be taken up by HepG_2_ cells, while cells showed significantly stronger fluorescence signal after incubated with coumarin-6-loaded NPs. These results indicate the excellent role of NUC-PLGA-NPs in the treatment of alleviating lipogenesis, for their perinuclear location might be important for higher drug concentration to address excess lipid accumulation within hepatocytes.

### Cytotoxicity and apoptosis of nanoparticles

**Figure 3.**
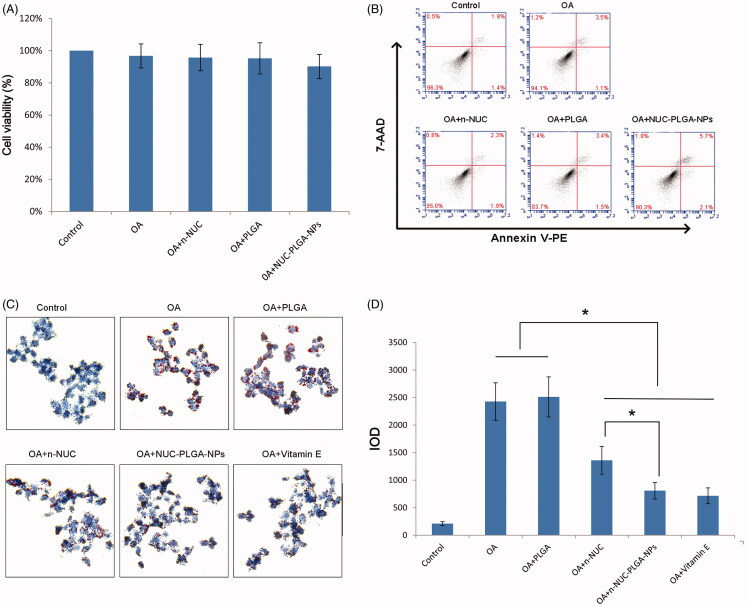
Cell viability (A) (mean ± SD, *n* = 3) and apoptosis (B) of HepG2 cells treated various formulations. (C) The effect of NUC on TG accumulated HepG2 of OA-induced hepatic steatosis. (D) Relative integrated option density (IOD) value was quantitated using Image Pro Plus 6.0 (mean ± SD, *n* = 3). *p < 0.05.

Prior to evaluating the reduction of the n-NUC and NUC-PLGA-NPs on lipid droplet accumulated in OA-induced hepatic steatosis, their impact on cell viability and apoptosis against HepG_2_ was tested in order to avoid any misinterpretation due to cytotoxicity. HepG_2_ cells incubated with n-NUC and NUC-PLGA-NPs supplemented with OA showed cell viability above 90% (Figure 3A). Concentration of OA at 0.04 mM and NUC at 0.05 mM reveals no significant difference compared with the control group. For better comparison among different drugs, OA at 0.04 mM and NUC at 0.05 mM both in n-NUC and NUC-PLGA-NPs were used for all the subsequent experiments.

Annexin V-PE/7AAD is used to quantitatively determine the percentage of cells undergoing apoptosis. As shown in Figure 3(B), few apoptotic cells were detected in the control group and no significant increase in apoptotic cells was observed in the cells treated with various formulations, suggesting that it is possible to model a liver steatosis with distinct lipid accumulation and without apparent cytotoxicity and apoptosis.

### NUC reduced TG accumulation in steatotic HepG_2_ cells

To examine the reduction of NUC on TG accumulation in OA-treated HepG_2_ cells, steatotic HepG_2_ cells were incubated with various treatments and evaluated using ORO staining. The steatosis was simulated *in vitro* by treating HepG_2_ cells with OA. Both OA, a monosaturated omega-9 fatty acid, and palmitic acid (PA), a saturated fatty acid, are the dominating dietary free fatty acids. Owing to the more steatogenic but less apoptotic nature of OA to hepatic cells than PA, OA was widely used to induce liver steatosis (Cui et al., 2016). As shown in Figure 3(C), intracellular lipid droplets were positively stained with ORO solution of OA-treated cells and negatively stained in cells without OA treatment. The low level of OA at 0.04 mM is able to cause prominent ORO-stained lipid droplets in the cytoplasm of cells, which might be due to the deficiency of communication between HepG2 and other types of cells, particularly adipocytes. As previously demonstrated, OA treatment exhibited a dose-dependent increase in lipid accumulation and cytotoxicity. Considering both the lipid accumulation and cytotoxicity, 0.04 mM was selected as the optimal concentration for the induction of lipid accumulation in HepG_2_ cells as a model of hepatic steatosis. As shown in Figure 3(D), TG was markedly accumulated in OA-treated cells compared with NCs. n-NUC and NUC-PLGA-NPs could decrease the intracellular TG content when compared to OA group (*p < 0.05). In addition, NUC-PLGA-NPs are more efficient against OA-induced TG accumulation, which was comparable with that of vitamin E and the percentage of inhibition is significantly higher than n-NUC (*p < 0.05) due to improved water solubility and sustained release. The first stage of non-alcoholic fatty liver disease (NAFLD) has nothing to do with excess alcohol consumption, but closely associated with the accumulation of lipids in hepatocytes. TG deposition, the primary pathological feature of liver steatosis, is regarded as the first stage in the evolution of NAFLD. In our previous study, we have demonstrated that NUC contributed to the inhibition of TG accumulation, while the better coordinating role of NUC-PLGA-NPs on TG over-accumulation has been demonstrated in this study.

### Pharmacokinetics and bioavailability *in vivo*

**Figure 4.**
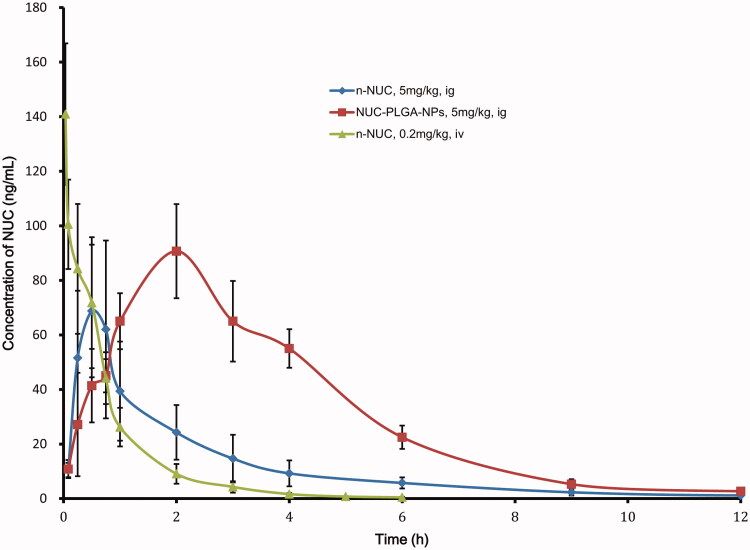
Mean plasma concentration–time curves of NUC (mean ± SD, *n* = 6).

The mean plasma concentration–time profiles of NUC after both the IV administration of NUC and the oral administration of n-NUC and NUC-PLGA-NPs are presented in Figure 4; the calculated pharmacokinetic parameters are summarized in Table 1 as well. After oral administration, the plasma concentrations of NUC-loaded NPs were all higher than that of the plain drug at nearly every time point. The t_1/2_ and t_max_ for NUC-PLGA-NPs was found to be 1.8 ± 0.2 and 1.3 ± 0.3 h, respectively, which was higher than n-NUC for which t_1/2_ was 0.8 ± 0.4 h and t_max_ was 0.6 ± 0.2 h. Moreover, the AUC_t_ for NUC-PLGA-NPs was extremely higher than n-NUC (**p < 0.01). The result showed that our NUC-PLGA-NPs had higher plasma concentration, lower clearance, and longer half-life as compared with NUC in rats. The lesser C_max_ for plain drug was due to self-aggregation, poor permeability and extensive metabolism mediated by P-glycoprotein (P-gp) efflux pump, while the improvement in C_max_ and AUC_t_ for NPs could be attributed to a decrease in first pass metabolism, as NPs reaches systemic circulation through gut-associated lymphatic tissue (Ahmad et al., 2015). The absolute bioavailability of NUC was a significant increase from 4.2 ± 1.3 to 13.5 ± 1.9. The relative bioavailability was increased by 3.3 ± 0.61-fold in NUC-PLGA-NPs as compared to corresponding n-NUC.

**Table 1.** Pharmacokinetic parameters of NUC (mean ± SD, *n* = 6).

Administration	Units	n-NUC, IV	n-NUC, IG	NUC-PLGA-NPs, IG
Dose	mg/kg	0.2	5	5
t_1/2_	h	0.6 ± 0.2	0.8 ± 0.4	1.8 ± 0.2^a^
C_max_	μg/mL	_	63.4 ± 19.0	77.9 ± 4.9
t_max_	min	_	0.6 ± 0.2	1.3 ± 0.3^a^
AUC_t_	min μg/mL	119 ± 34.1	118.2 ± 52.5	400.2 ± 64.4^b^
F_abs_	%	_	4.2 ± 1.3	13.5 ± 1.9^b^
F_rel_	%	_	_	330 ± 61

t_1/2_: half-life time; C_max_: peak concentration; t_max_: time to reach C_max_; AUC_t_: total area under curve; F_abs_: absolute bioavailability; F_rel_: relative bioavailability; NUC: nuciferine; IV: intravenous administration; IG: intragastric administration; NPs: nanoparticles

^a^ *p *< 0.05 and ^b^*p *< 0.01 versus n-NUC

### Serum biochemical parameters

The serum biochemical parameters after 2, 4, 6 and 8 weeks’ treatment are listed in Table 2. The HC group exhibited significantly increased serum levels of TC, TG and LDL-C, as well as lower concentration of HDL-C, compared with that of NC group. These results demonstrated that the HC group exhibited dyslipidemia to some extent. However, the abnormal serum lipid levels were prevented by NUC and the treatment of NUC-PLGA-NPs was better than n-NUC. The administration of NUC-PLGA-NPs reduced serum TC and HDL-C levels compared with that of HC group after 2 weeks (**p < 0.01), and even more significantly after 6 weeks (***p < 0.001). n-NUC also showed significant effects on HDL-C levels after 2 weeks (**p < 0.01) and TC levels after 6 weeks (*p <0.05). In addition, the level of TG and LDL-C decreased only when rats treated NUC-PLGA-NPs after 8 weeks (*p <0.05). In this research, the intervention of both NUC and NUC-PLGA-NPs might prevent hyperlipidemia from HFD rats, and NUC-PLGA-NP treatment plays a significant role in regulating blood lipids due to the improved bioavailability, sustained and controlled release. As a broad term containing various lipid and/or lipoprotein disorder, the primary manifestations of dyslipidemia are the increased serum lipid concentrations of TC, TG and LDL-C, as well as the low concentrations of HDL-C. TC and TG are the dominating lipids circulating in the blood. The danger of increased TC was clear. The role of TC levels in various diseases was controversial, while high levels of TC are often related to metabolic syndrome and always accompanied with low levels of good cholesterol named HDL-C, which induced vascular disease (Zhou et al., 2016). The excessive LDL-C, bad cholesterol, will initiate the formation of atherosclerotic plaques (Gao et al., 2014). Therefore, high levels of TC, TG and LDL-C, as well as low levels of HDL-C in the blood could be regarded as a danger sign.

**Table 2.** Serum indexes for male Sprague Dawley rats after 2, 4, 6 and 8 weeks of treatment (mean ± SD, *n* = 6).

		Serum indexes (mmol/L)			
Time	Group	TC	TG	HDL-C	LDL-C
2 weeks	NC	1.64 ± 0.13	0.44 ± 0.20	2.12 ± 0.22	0.36 ± 0.14
	HC	2.78 ± 0.18	0.42 ± 0.13	1.41 ± 0.23	0.51 ± 0.12
	n-NUC	2.56 ± 0.22	0.45 ± 0.13	1.99 ± 0.26^b^	0.52 ± 0.25
	NUC-PLGA-NPs	2.33 ± 0.22^b^	0.45 ± 0.11	2.00 ± 0.26^b^	0.46 ± 0.27
4 weeks	NC	1.64 ± 0.23	0.38 ± 0.20	2.12 ± 0.27	0.40 ± 0.24
	HC	2.55 ± 0.13	0.51 ± 0.20	1.44 ± 0.17	0.52 ± 0.16
	n-NUC	2.50 ± 0.17	0.47 ± 0.15	1.99 ± 0.32^b^	0.52 ± 0.09
	NUC-PLGA-NPs	2.2 ± 0.21^b,d^	0.41 ± 0.19	2.03 ± 0.30^b^	0.39 ± 0.15
6 weeks	NC	1.68 ± 0.29	0.40 ± 0.16	2.08 ± 0.16	0.40 ± 0.93
	HC	2.74 ± 0.19	0.48 ± 0.26	1.39 ± 0.13	0.45 ± 0.11
	n-NUC	2.40 ± 0.22^a^	0.41 ± 0.18	1.63 ± 0.206^a^	0.54 ± 0.08
	NUC-PLGA-NPs	2.11 ± 0.22^c,d^	0.41 ± 0.20	2.06 ± 0.27^c,d^	0.49 ± 0.20
8 weeks	NC	1.72 ± 0.30	0.40 ± 0.22	2.03 ± 0.28	0.39 ± 0.17
	HC	2.8 ± 0.18	0.54 ± 0.20	1.34 ± 0.26	0.48 ± 0.13
	n-NUC	1.93 ± 0.26^c^	0.45 ± 0.18	1.76 ± 0.18^b^	0.47 ± 0.20
	NUC-PLGA-NPs	1.38 ± 0.15^c,f^	0.30 ± 0.13^d^	2.17 ± 0.24^c,e^	0.31 ± 0.11^d^

NC: normal control; HC: hyperlipidemia control; NUC: nuciferine; NPs: nanoparticles; TC: total cholesterol; TG: triglycerides; HDL-C: high density lipoprotein cholesterol; LDL-C: low-density lipoprotein cholesterol

^a^ *p *< 0.05, ^b^*p *< 0.01 and ^c^*p *< 0.001 versus HC in various weeks, respectively.

^d^ *p *< 0.05, ^e^*p *< 0.01 and ^f^*p *< 0.001 versus n-NUC in various weeks, respectively.

## Conclusion

In summary, the s/o/w emulsion technique selected in this research allowed the reproducible and momentary formation of nanometric (∼150 nm), homogeneous and spherical nanocomposites, which exhibited a remarkable loading capacity for NUC with an adjustable dosage ratio. The oral bioavailability of NUC-PLGA-NPs was significantly increased to 3.3 ± 0.61-fold when compared to that of n-NUC in rats. In addition, NUC-PLGA-NPs are more efficient in alleviating lipogenesis in both *in vitro* and *in vivo* evaluation due to the improved water solubility and prolonged incubation time. These findings indicated that NUC-PLGA-NPs hold great promise for sustained and controlled drug delivery with improved bioavailability to alleviating lipogenesis.
